# DEPDC1B: A novel tumor suppressor gene associated with immune infiltration in colon adenocarcinoma

**DOI:** 10.1002/cam4.70043

**Published:** 2024-08-01

**Authors:** Dandan Zhu, Huolun Feng, Zhixiong Zhang, Jiaqi Li, Yong Li, Tieying Hou

**Affiliations:** ^1^ Guangdong Center for Clinical Laboratory, Guangdong Provincial People's Hospital (Guangdong Academy of Medical Sciences) Southern Medical University Guangzhou Guangdong China; ^2^ School of Medicine South China University of Technology Guangzhou Guangdong China; ^3^ Department of Gastrointestinal Surgery, Department of General Surgery, Guangdong Provincial People's Hospital (Guangdong Academy of Medical Sciences) Southern Medical University Guangzhou Guangdong China; ^4^ Medical Experimental Center Shenzhen Nanshan People's Hospital Shenzhen Guangdong China; ^5^ Medical School Shenzhen University Shenzhen Guangdong China

**Keywords:** biomarker, cell cycle, colon adenocarcinoma, immunotherapy, tumor‐infiltrating immune cells

## Abstract

**Background:**

Recent research indicates a positive correlation between DEP structural domain‐containing 1B (DEPDC1B) and the cell cycle in various tumors. However, the role of DEPDC1B in the infiltration of the tumor immune microenvironment (TIME) remains unexplored.

**Methods:**

We analyzed the differential expression and prognostic significance of DEPDC1B in colon adenocarcinoma (COAD) using the R package “limma” and the Gene Expression Profiling Interactive Analysis (GEPIA) website. Gene set enrichment analysis (GSEA) was employed to investigate the functions and interactions of DEPDC1B expression in COAD. Cell Counting Kit‐8 (CCK‐8) assays and colony formation assays were utilized to assess the proliferative function of DEPDC1B. Correlations between DEPDC1B expression and tumor‐infiltrating immune cells, immune checkpoints, tumor mutational burden (TMB), and microsatellite instability (MSI) status were examined using Spearman correlation analysis and CIBERSORT.

**Results:**

DEPDC1B was highly expressed in COAD. Elevated DEPDC1B expression was associated with lower epithelial‐to‐mesenchymal transition (EMT) and TNM stages, leading to a favorable prognosis. DEPDC1B mRNA was prominently expressed in COAD cell lines. CCK‐8 and colony formation assays demonstrated that DEPDC1B inhibited the proliferation of COAD cells. Analysis using the CIBERSORT database and Spearman correlation revealed that DEPDC1B correlated with four types of tumor‐infiltrating immune cells. Furthermore, high DEPDC1B expression was linked to the expression of PD‐L1, CTLA4, SIGLEC15, PD‐L2, TMB, and MSI‐H. High DEPDC1B expression also indicated responsiveness to anti‐PD‐L1 immunotherapy.

**Conclusions:**

DEPDC1B inhibits the proliferation of COAD cells and positively regulates the cell cycle, showing a positive correlation with CCNB1 and PBK expression. DEPDC1B expression in COAD is associated with tumor‐infiltrating immune cells, immune checkpoints, TMB, and MSI‐H in the tumor immune microenvironment. This suggests that DEPDC1B may serve as a novel prognostic marker and a potential target for immunotherapy in COAD.

## 1 INTRODUCTION

Colorectal cancer stands out as the most prevalent form of cancer globally, boasting the highest rates of both incidence and mortality.^1^ Among the various tissue subtypes of colon cancer, colon adenocarcinoma (COAD) takes precedence as the most common. Notably, the prognosis for individuals with locally advanced colon cancer has seen substantial enhancement with available treatments,^2^, ^3^, ^4^ with immunotherapy, in particular, playing a significant role in this improvement.^5^, ^6^, ^7^ Consequently, the quest for markers associated with immunotherapy hold paramount importance in the realm of targeted immunotherapy.

Aberrant expression of DEPDC1B is implicated in the development of various tumor types, as highlighted in multiple studies.^8^, ^9^, ^10^, ^11^, ^12^, ^13^, ^14^, ^15^, ^16^ For instance, DEPDC1B regulates the progression of human chordoma by influencing the ubiquitination of baculovirus apoptosis repeat inhibitor 5 (BIRC5) through the ubiquitin‐binding enzyme E2T (UBE2T);^8^ in bladder cancer, DEPDC1B and SHC1 collectively serve as tumor promoters, impacting cell proliferation, apoptosis, and migration;^9^ DEPDC1B regulates the development of hepatocellular carcinoma by controlling cell proliferation, apoptosis, the cell cycle, and cell migration. It interacts with CDK1, playing a crucial role in DEPDC1B's regulation of hepatocellular carcinoma progression;^10^ inducing EMT through the activation of the Akt/GSK3β/Snail pathway, DEPDC1B promotes migration and invasion of pancreatic ductal adenocarcinoma cells;^14^ in prostate cancer, DEPDC1B induces EMT and enhances proliferation and migration through the Rac1‐PAK1 signaling pathway by interacting with Rac1;^11^ acting downstream of SOX10, DEPDC1B promotes melanoma angiogenesis and metastasis by isolating the stably secreted SCUBE3 through CDC16;^12^ and DEPDC1B promotes invasion and metastasis of non‐small cell lung cancer in a Wnt/β‐catenin‐dependent manner.^17^ In conclusion, the aforementioned studies collectively demonstrate the significant role played by DEPDC1B in cancer development through the activation of diverse downstream events. While a recent study on colorectal cancer revealed that DEPDC1B promotes the proliferation and migration of colorectal cancer cells while inhibiting apoptosis,^18^ the role of DEPDC1B in the tumor immune microenvironment of COAD has yet to be investigated.

In the current investigation, our findings revealed high expression of DEPDC1B in COAD tissues, and this elevated expression was associated with a favorable prognosis. Results from GSEA indicated a correlation between high DEPDC1B expression and the cell cycle. Furthermore, CCK‐8 assays and colony formation assays suggested that the knockdown of DEPDC1B promoted the proliferation of COAD cells. Utilizing the CIBERSORT algorithm and Spearman correlation analysis, we observed a positive correlation between DEPDC1B expression and the infiltration of T‐cells follicular helper (Tfh) cells, while a negative correlation was identified with T‐cells regulatory (Treg) infiltration. Lastly, Spearman correlation analysis demonstrated a positive correlation between DEPDC1B expression and the expression of immune checkpoints. Notably, high DEPDC1B expression was linked to a positive response to immunotherapy involving PD‐L1. In summary, our study suggests that DEPDC1B could be a potential target for immunotherapy in the context of COAD.^19^

## 2 MATERIALS AND METHODS

### 2.1 Data collection

We utilized the Genomics Data Commons Data Transfer Tool (https://gdc.cancer.gov/access‐data/gdc‐data‐transfer‐tool.html) to download the published data of the Cancer Genome Atlas (TCGA) RNA‐seq data and the corresponding clinical information for COAD. The screening criteria for mRNAs comprised “Project: TCGA‐COAD,” “Experimental strategy: RNA‐Seq,” and “Workflow type: HTSeq‐Counts,” encompassing a total of 398 COAD tissues and 39 normal colon tissues. Additionally, clinical follow‐up datasets from 386 patients with COAD were acquired from the TCGA database.^19^

### 2.2 Differential expression, prognosis and related gene analysis of DEPDC1B

We categorized the 398 patients with COAD into DEPDC1B‐high expression and DEPDC1B‐low expression groups based on the median expression values of DEPDC1B. Utilizing the R packages “limma” and “survival”, we conducted differential expression and survival analyses of DEPDC1B on COAD tissues and their corresponding paraneoplastic tissues, respectively. Following the grouping of the 398 COAD patients based on DEPDC1B expression, we identified differentially expressed genes and performed correlation analysis. Subsequently, genes exhibiting the highest correlation with DEPDC1B were identified and validated using Spearman correlation analysis. Additionally, we conducted further analyses, including differential expression analysis and survival analysis, on these identified genes.

### 2.3 Gene set enrichment analysis (GSEA)

GSEA is a computational method used to assess whether a pre‐defined set of genes exhibits statistical significance between two biological expression states. In this study, we selected “c2.cp.kegg.v7.4.symbols.gmt” from the Molecular Signatures Database (MSigDB) as the reference gene set for conducting GSEA between the DEPDC1B high and low expression groups. To address the challenge of multiple hypothesis testing, we calculated false discovery rate (FDR) adjusted *p*‐values (*q*‐values) in the GSEA analysis. We utilized the sequential *p*‐values approach proposed by Benjamini and Hochberg.^20^ A *q*‐value below 0.05 was considered statistically significant. Finally, we presented the top five pathways that demonstrated significant enrichment and statistical significance.

### 2.4 Cell lines and cell culture

The COAD cell lines, including HCT116, RKO, HCT15, HCT8, DLD‐1, HT29, and the human colonic epithelial cell line (NCM460), were procured from the American Type Culture Collection (ATCC), located in Manassas, VA, USA. All COAD cell lines were cultured in 1640 medium (Gibco, Gaithersburg, MD, USA) supplemented with 10% fetal bovine serum (FBS, Gibco‐BRL, Paisley, UK), 100 U/mL penicillin, and 100 μg/mL streptomycin. The cultures were maintained at 37°C in an environment containing 5% CO_2_.

### 2.5 RNA extraction and quantitative real‐time PCR (qRT‐PCR) and reverse transcription

Total RNA extraction from cell lines was carried out using TRIzol® Reagent (Invitrogen, Carlsbad, CA, USA). Subsequently, the obtained total RNA underwent reverse transcription into cDNA using PrimeScript™ RT Master Mix (TaKaRa, Dalian, China). The resulting cDNA was then employed for qRT‐PCR using SYBR® qPCR Master Mix (Vazyme, Nanjing, China). Glyceraldehyde 3‐phosphate dehydrogenase (GAPDH) served as the internal control for gene quantification. The 2^(−ΔCT)^ value was calculated for each sample and normalized to the corresponding GAPDH value. The primer sequences utilized for PCR are provided in Table S1.

### 2.6 RNA‐seq analysis

Total RNA from DEPDC1B knockdown or control HCT116 cells was isolated and purified using TRIzol (Invitrogen, CA, USA), following the manufacturer's protocol. RNA quantification and quality control were conducted using an Agilent Bioanalyzer 2100 (Agilent, CA, USA). For high‐throughput sequencing, stranded RNA‐seq libraries were constructed using the NEBNext® UltraTM RNA Library Prep Kit (NEB, USA), following the manufacturer's protocol. Indexing of the reference genome was established using HISAT2 v2.0.5, and paired clean reads were aligned to the reference genome using HISAT2 v2.0.5 and RseQC. StringTie software was employed for the primary assembly of genes or transcripts. The primary assembly results from all samples were combined, and gffcompare software was used to detect transcript comparisons with the reference annotation, yielding the final assembly annotation results. RSEM was utilized to calculate the number of reads mapped to each gene/transcript level and the Fragments Per Kilobase Million (FPKM) of each gene. Differential expression analysis was carried out using DEGseq version 1.36.1, with *p*‐values calculated using Student's *t*‐test. Statistical enrichment analysis of differentially expressed genes in the Kyoto Encyclopedia of Genes and Genomes (KEGG) pathway was performed using the R package “clusterProfiler.”^21^, ^22^, ^23^

### 2.7 Cell Counting Kit‐8 (CCK‐8) assays

CCK‐8 (Dojindo Laboratories Kumamoto, Japan) assays were employed for cell proliferation analysis following the manufacturer's instructions. Cells were cultured in each well of a 96‐well plate at a density of 2 × 10^3^ cells/well. Subsequently, 100 μL of CCK‐8 solution (prepared by mixing 10 μL of CCK‐8 reagent with 100 μL of culture medium) was added to each well at various time points (24, 48, 72, and 96 h). The absorbance was measured at 450 nm after incubation at 37°C for 2 h.^19^

### 2.8 Colony formation assays

We evenly seeded 1000 cells in each well of six‐well plates and cultured them for 14 days. Following colony formation, we added 4% paraformaldehyde for fixation, allowing it to set for 30 to 60 min. Subsequently, the cells were stained with crystal violet for 10 to 20 min. Finally, the cell formations in each well were photographed using a digital camera.

### 2.9 CIBERSORT estimation and immune‐related analysis

We then investigated whether DEPDC1B influences the immune microenvironment. To do so, we analyzed immune‐infiltrating cells with significant differences between the DEPDC1B high and low expression groups using the Wilcoxon rank sum test. Spearman correlation analysis was conducted for DEPDC1B and the proportion of each related immune cell with *p* < 0.05. The immune cells that exhibited differential expression in the high and low DEPDC1B groups were overlapped with immune cells associated with DEPDC1B expression using the R package “VennDiagram.” This allowed us to identify immune cells specifically associated with DEPDC1B. To explore the potential role of DEPDC1B in immunotherapy, we assessed the correlation of DEPDC1B expression with immune checkpoints, TMB, and MSI status using Spearman correlation analysis.

## 3 RESULTS

### 3.1 DEPDC1B is upregulated in COAD and associated with a better prognosis

**FIGURE 1:**
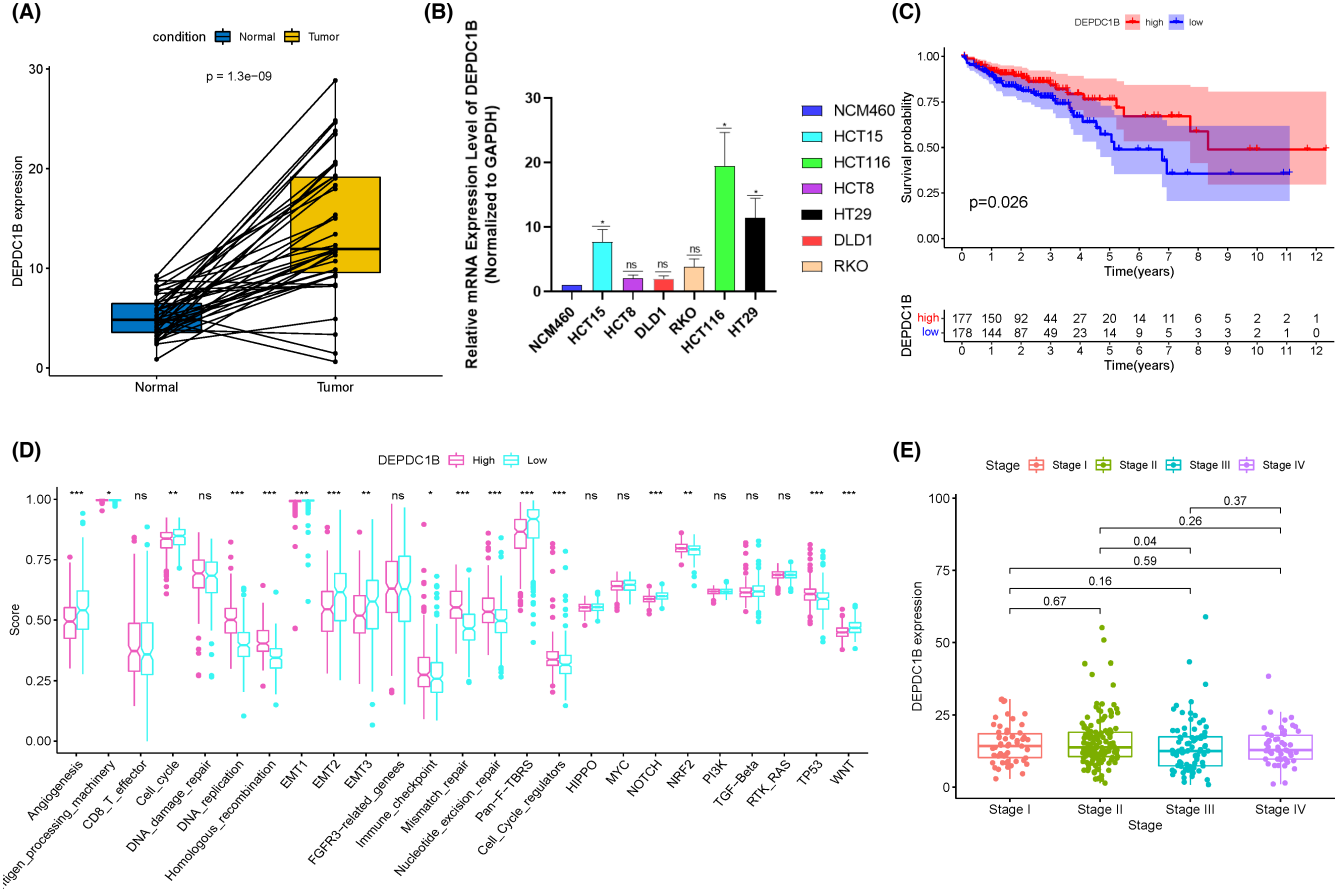
Differential expression of DEPDC1B in COAD and its biological role. (A) The mRNA expression of DEPDC1B is significantly upregulated in COAD tissues compared with normal colon tissues. (B) qRT‐PCR validation of DEPDC1B expression in COAD cells compared to control cells. (C) Survival curves of TCGA data stratified by DEPDC1B mRNA expression. (D) Scoring of DEPDC1B in pathways related to biological actions. (E) Expression levels of DEPDC1B in different TNM stages of COAD patients. *p < 0.05, **p < 0.01, ***p < 0.001.

DEPDC1B exhibited significantly elevated expression in COAD tissues compared to normal colon tissues (*p* = 1.3e−09) (Figure 1A). qRT‐PCR analysis further confirmed the heightened expression of DEPDC1B in the majority of COAD cell lines in comparison to control cells (NCM‐460) (Figure 1B). Survival analysis indicated a higher overall survival rate among patients with high DEPDC1B expression (*p* = 0.034) (Figure 1C). Examining the biological role of DEPDC1B, high expression was associated with low levels of angiogenesis, cell cycle activity, EMT, pan‐FTBRS, and TNM staging, consequently correlating with a favorable prognosis (Figure 1D). Additionally, DEPDC1B expression exhibited relatively lower levels in COAD patients with higher TNM staging (Figure 1E).

### 3.2 DEPDC1B expression was positively correlated with CCNB1 and PBK expression

**FIGURE 2:**
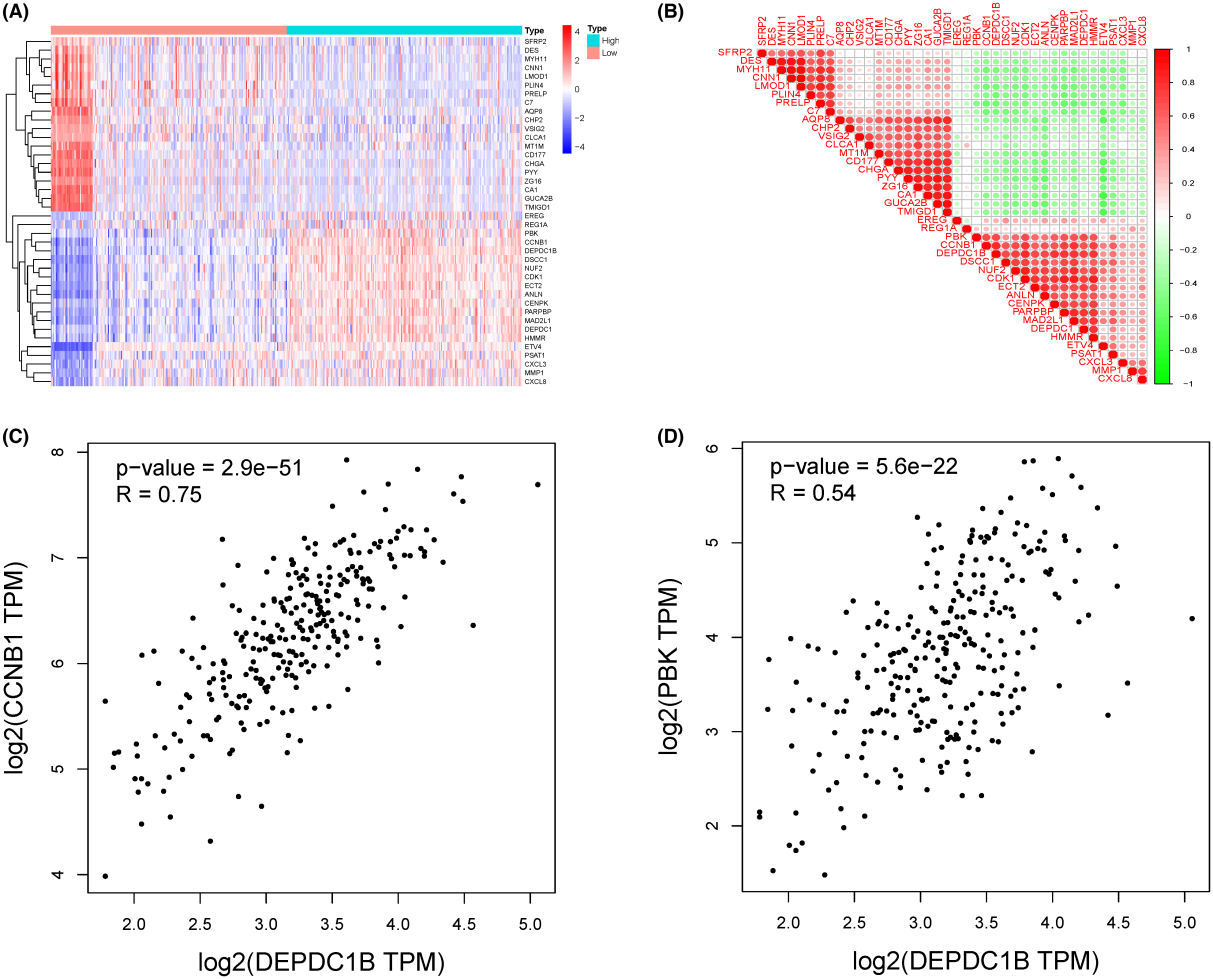
Genes closely associated with DEPDC1B expression and their analysis. (A) Genes differentially expressed between the high and low DEPDC1B expression groups. (B) Correlation analysis of differentially expressed genes between the high and low DEPDC1B expression groups. Correlation analysis of DEPDC1B with (C) CCNB1 and (D) PBK.

We identified differentially expressed genes between the DEPDC1B high and low expression groups (Figure 2A) and conducted correlation analysis on these genes. Among them, CCNB1 and PBK exhibited the highest correlation with DEPDC1B (Figure 2B–D). The expression patterns and prognostic trends of CCNB1 and PBK mirrored those of DEPDC1B (Figure S1).

### 3.3 Pathways enriched between the high and low DEPDC1B expression groups

**FIGURE 3:**
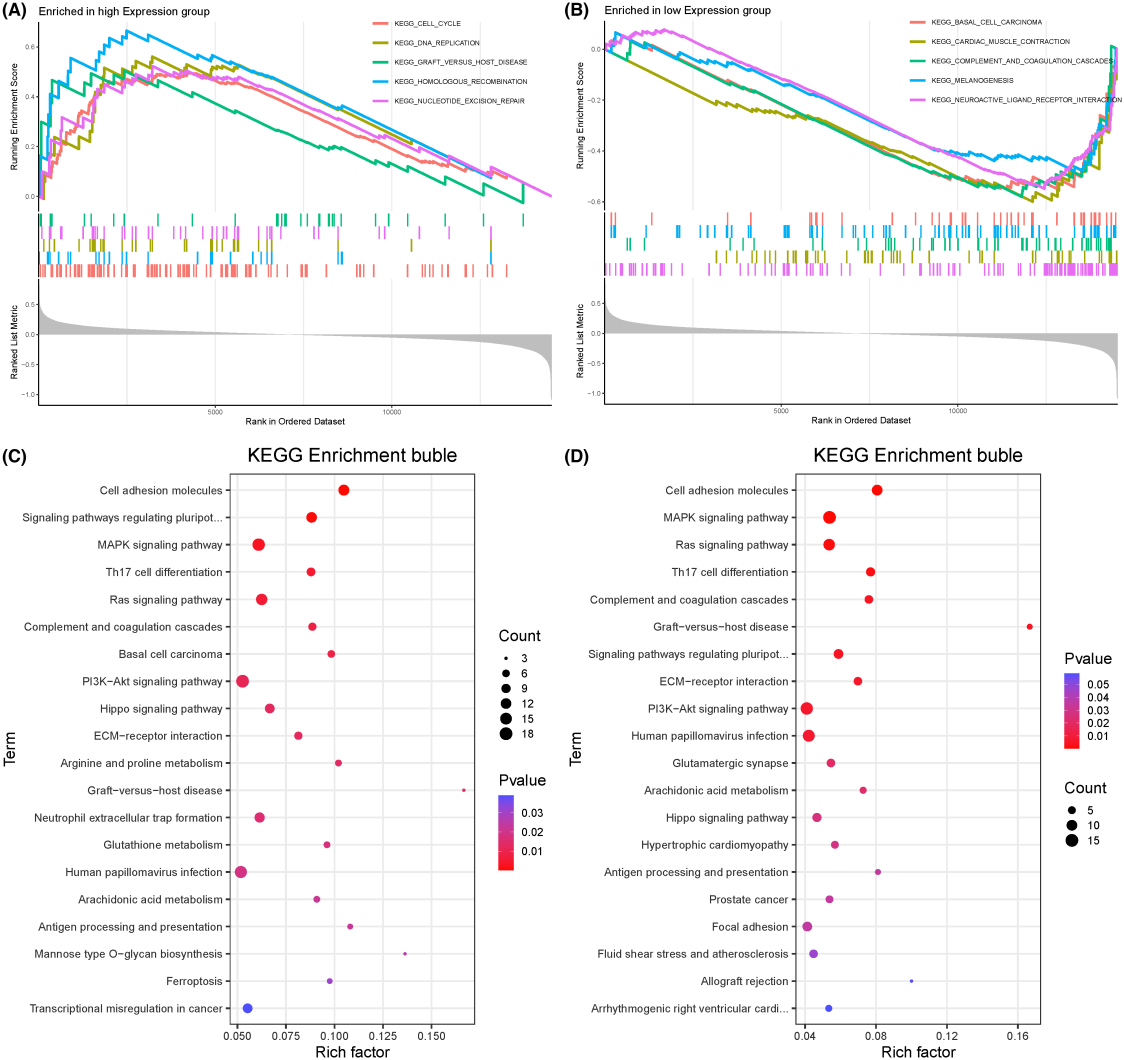
Pathways enriched between the high and low DEPDC1B expression groups. Upregulated gene sets in the DEPDC1B (A) high and (B) low expression groups using gene set enrichment analysis (GSEA). Kyoto Encyclopedia of Genes and Genomes (KEGG) analysis of (C) upregulated and (D) downregulated genes in DEPDC1B knockdown HCT116 cells.

According to the GSEA results, in the DEPDC1B high expression group, the top five enriched pathways were “CELL_CYCLE”, “DNA_REPLICATION,” “GRAFT_VERSUS_HOST_DISEASE,” “HOMOLOGOUS_RECOMBINATION,” and “NUCLEOTIDE_EXCISION_REPAIR” (Figure 3A). In contrast, the DEPDC1B low expression group exhibited enrichment in the pathways “BASAL_CELL_CARCINOMA,” “CARDIAC_MUSCLE_CONTRACTION,” “COMPLEMENT_AND_COAGULATION_CASCADES,” “MELANOGENESIS,” and “NEUROACTIVE_LIGAND_RECEPTOR_INTERACTION” (Figure 3B). To further validate the pathways with differential enrichment between the high and low DEPDC1B expression groups, we conducted RNA‐seq on DEPDC1B knockdown HCT116 cells. A total of 443 genes were upregulated, and 601 genes were downregulated in the DEPDC1B knockdown HCT116 cells compared with the control HCT116 cells. KEGG pathway enrichment analyses were performed on the up‐ and down‐regulated genes between the DEPDC1B knockdown and control groups, and the KEGG results (Figure 3C,D) were consistent with those observed in the GSEA.

### 3.4 DEPDC1B knockdown promotes the proliferation of COAD cells

**FIGURE 4:**
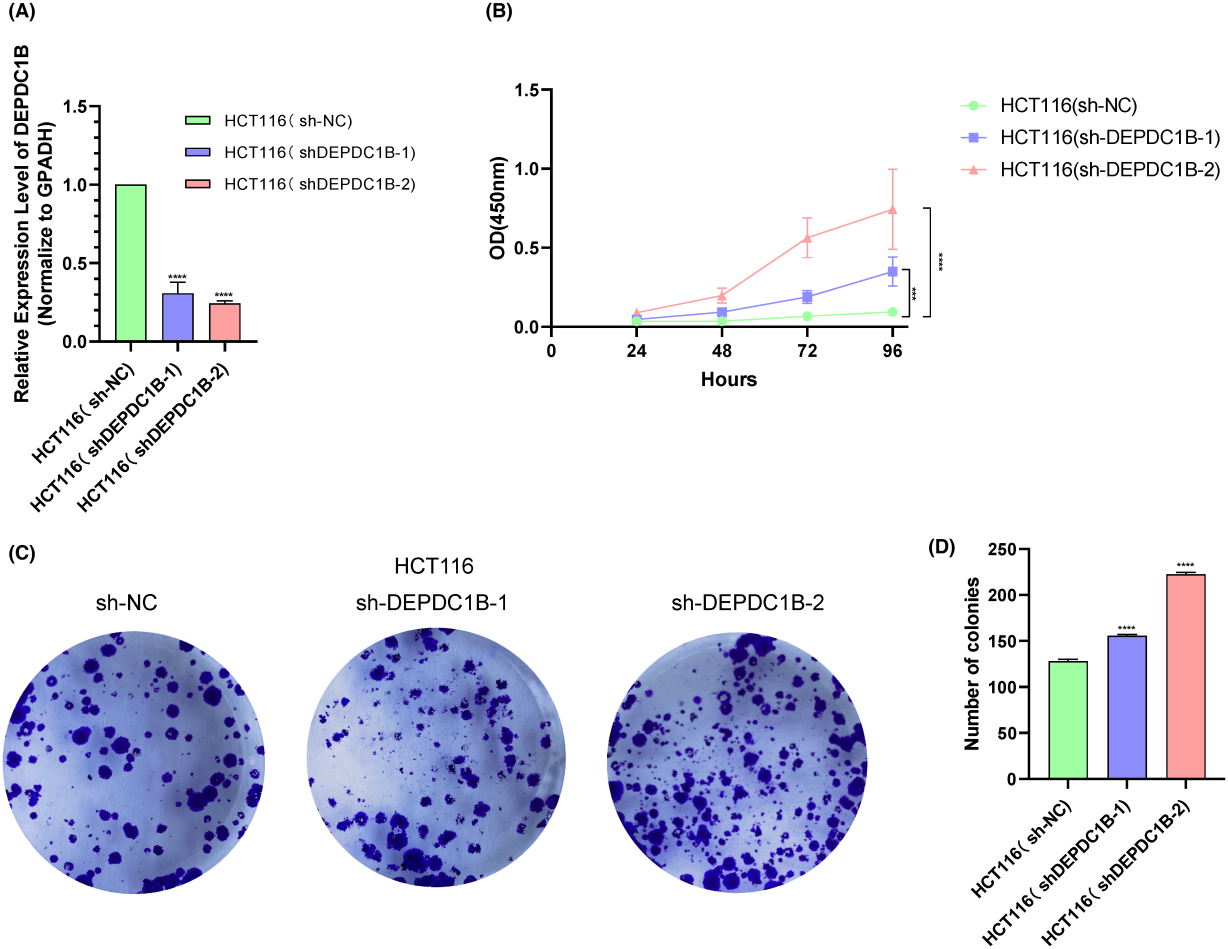
Knockdown of DEPDC1B promotes the proliferation of HCT116 cells. (A) Effect of knockdown of DEPDC1B expression in the HCT116 cells after transfection with shRNA. The effects of DEPDC1B knockdown on the proliferation of the HCT116 cells were examined by (B) CCK‐8 assays and (C‐D) colony formation assays. ***p < 0.001, ****p < 0.0001.

We chose HCT116 cells with high DEPDC1B expression, commonly utilized for studying the COAD phenotype, for the CCK‐8 and colony formation assays. The expression of DEPDC1B in HCT116 cells was effectively reduced by establishing stable cell lines through shRNA infection (Figure 4A). The results of the CCK‐8 assays revealed a significant increase in the proliferation of HCT116 cells with DEPDC1B knockdown compared to the control group (Figure 4B). Additionally, colony formation assays demonstrated that DEPDC1B knockdown led to an augmentation in the colony formation of HCT116 cells (Figure 4C,D).

### 3.5 The composition of tumor‐infiltrating immune cells correlated with DEPDC1B expression

**FIGURE 5:**
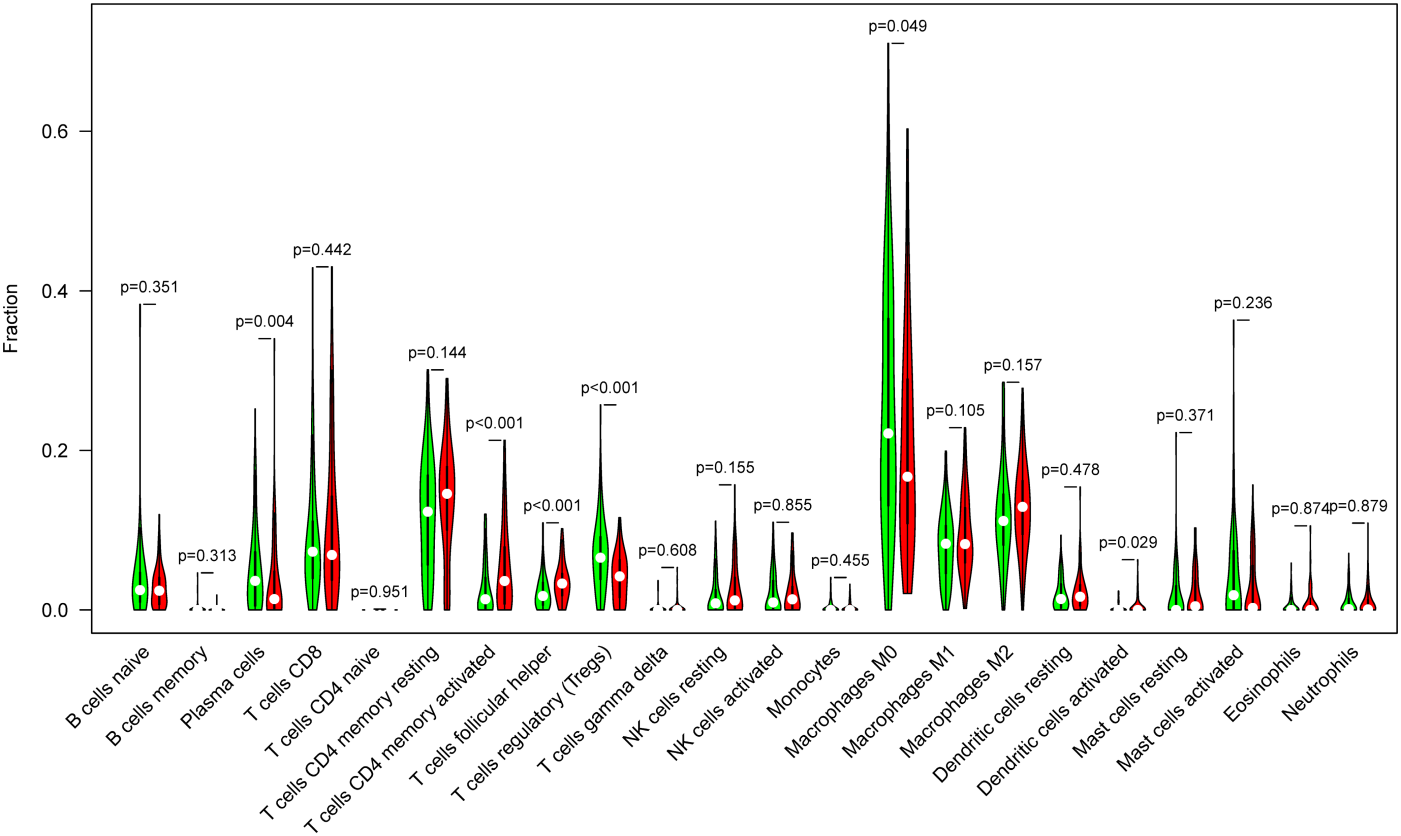
Significant tumor‐infiltrating immune cells were found between the high and low DEPDC1B expression groups in COAD samples.

**FIGURE 6:**
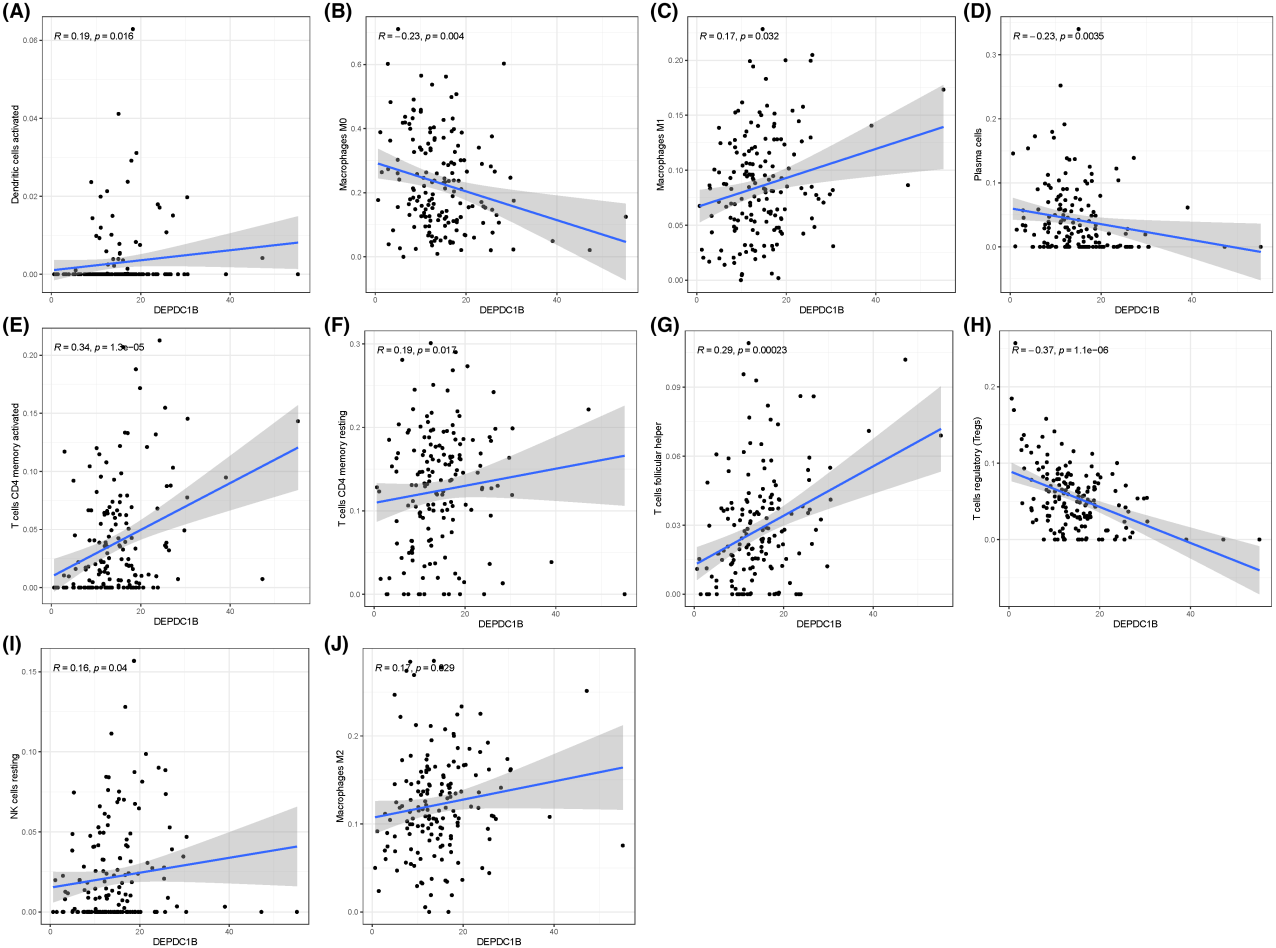
Significant correlation between DEPDC1B expression and immune cells. Significant correlations were found between DEPDC1B expression and immune cells including (A) plasma cells, (B) resting memory CD4 T cells, (C) activated memory CD4 T cells, (D) follicular helper T cells (Tfhs), (E) regulatory T cells (Tregs), (F) resting NK cells, (G) M0 macrophages, (H) M1 macrophages, (I) M2 macrophages and (J) activated dendritic cells.

We evaluated the composition of significant tumor‐infiltrating immune cells in COAD tissues using the CIBERSORT algorithm. The results from the Wilcoxon rank sum test indicated that the proportions of plasma cells (*p* < 0.005), activated memory CD4 T cells (*p* < 0.001), Tfhs (*p* < 0.001), Tregs (*p* < 0.001), M0 macrophages (*p* < 0.05), and activated dendritic cells (*p* < 0.05) differed significantly between the high and low DEPDC1B expression groups (Figure 5). Further investigation into the correlation between DEPDC1B expression and tumor‐infiltrating immune cells using Spearman correlation analysis revealed significant associations with various immune cell types. These included plasma cells (*p* < 0.005, cor = −0.23), resting memory CD4 T cells (*p* < 0.05, cor = 0.19), activated memory CD4 T cells (*p* < 0.001, cor = 0.34), Tfhs (*p* < 0.001, cor = 0.29), Tregs (*p* < 0.001, cor = −0.37), resting NK cells (*p* < 0.05, cor = 0.16), M0 macrophages (*p* < 0.005, cor = −0.23), M1 macrophages (*p* < 0.05, cor = 0.17), M2 macrophages (*p* < 0.05, cor = 0.17), and activated dendritic cells (*p* < 0.05, cor = 0.19) (Figure 6). Intersection of 10 differentially expressed immune cells with six related immune cells revealed five immune cells that were associated with DEPDC1B expression, including plasma cells, activated memory CD4 T cells, Tfhs, Tregs, M0 macrophages, and activated dendritic cells (Figure S2).

### 3.6 Single‐cell analysis of the expression of DEPDC1B in different immune cells of COAD

**FIGURE 7:**
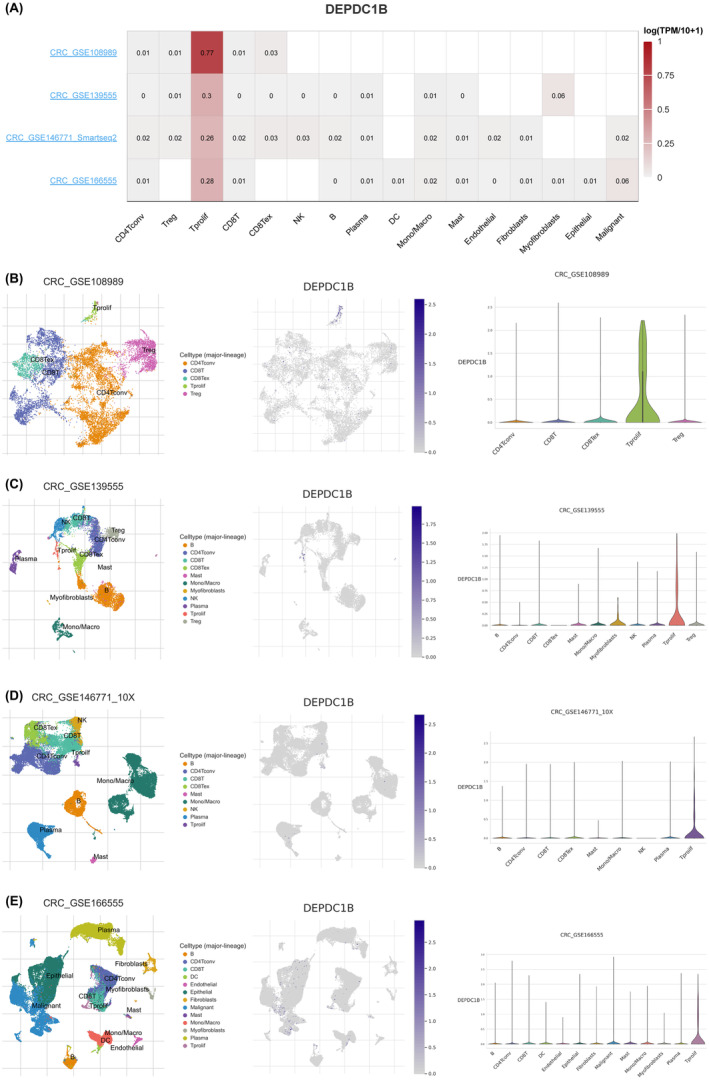
Single‐cell analysis of the expression of DEPDC1B in different immune cells of COAD. (A) Heatmap of the correlation of DEPDC1B with immune cell infiltration levels in four independent datasets based on the scRNA‐seq database. Violin plot and single‐cell atlas of NMUR1 and immune cell infiltration in (B) CRC_GSE108989, (C) CRC_GSE139555, (D) CRC_GSE146771 and (E) CRC_GSE166555.

Based on the scRNA‐seq TISCH database, we obtained four independent datasets of COAD (CRC_GSE108989, CRC_GSE139555, CRC_GSE146771 and CRC_GSE166555) for single‐cell sequencing to explore the correlation of immune cell distribution with DEPDC1B expression levels at the single‐cell level (Figure 7A). In the four datasets, DEPDC1B was expressed at high levels in Tprolif cells (Figure 7A–E). Tprolif cells can enhance or inhibit the function of other immune cells, thereby regulating the extent and direction of immune responses. Therefore, DEPDC1B expression levels may influence other immune cell types and their ratios through Tprolif cells, thereby affecting immunotherapy responses.

### 3.7 DEPDC1B expression is associated with immunotherapy

**FIGURE 8:**
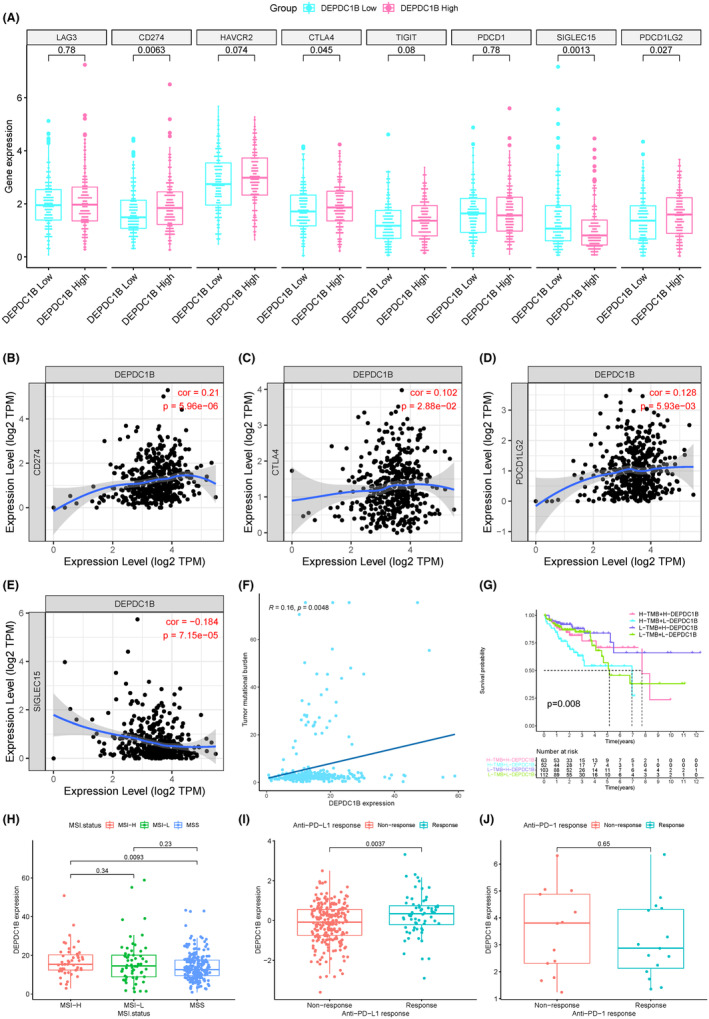
Analysis of DPEDC1B with immunotherapy and related markers. (A) Immune checkpoints differentially expressed between high and low DEPDC1B expression groups. Correlation analysis of DEPDC1B with (B) PD‐L1 (CD274), (C) CTLA4, (D) PD‐L2 (PDCD1LG2), (E) SIGLEC15 and (F) tumor mutation burden (TMB). (G) Survival curves of TCGA data stratified by DEPDC1B mRNA expression and high or low tumor mutation burden. (H) Expression of DEPDC1B between different microsatellite instability (MSI) statuses. Expression of DEPDC1B in the (I) anti‐PD‐L1 and (J) anti‐PD‐1 clinical response groups.

As shown in Figure 1D, DEPDC1B expression influenced immune checkpoint function. Analysis of the expression of current immune checkpoints between the high and low DEPDC1B expression groups revealed differential expression of PD‐L1 (CD274), CTLA4, SIGLEC15, and PD‐L2 (PDCD1LG2) (Figure 8A). Spearman correlation analysis of these immune checkpoints demonstrated a positive correlation between DEPDC1B expression and PD‐L1 (*p* = 5.96e−06, cor = 0.21), CTLA4 expression (*p* = 2.88e−02, cor = 0.102), and PD‐L2 (*p* = 5.93e−03, cor = 0.128) (Figure 8B–D), while it exhibited a negative correlation with SIGLEC15 (*p* = 7.15e−05, cor = −0.184) (Figure 8E). Spearman correlation analysis revealed a positive correlation between DEPDC1B and TMB (Figure 8F), and survival analysis indicated that COAD patients with high DEPDC1B and high TMB had a better prognosis (Figure 8G). Furthermore, DEPDC1B expression was lower in COAD patients with Microsatellite Stable (MSS) status compared to those with Microsatellite Instability‐High (MSI‐H) status (Figure 8H).

In the anti‐PD‐L1 cohort (IMvigor210 cohort), patients with high DEPDC1B expression exhibited a positive response to anti‐PD‐L1 treatment (*p* = 0.0037) (Figure 8I). However, in the anti‐PD‐1 cohort (GSE78220 cohort), DEPDC1B expression did not significantly correlate with the response to anti‐PD‐1 treatment (*p* = 0.65) (Figure 8J), suggesting that DEPDC1B may serve as a predictive marker for anti‐PD‐L1 therapy. In summary, these results suggest that DEPDC1B may play a role in the immune response within the tumor immune microenvironment.

## 4 DISCUSSION

COAD poses a significant global health challenge due to its high incidence and mortality rates, largely attributed to its resistance to current treatments.^1^ Consequently, gaining insights into the oncogenic drivers and understanding their clinical implications is crucial for developing effective treatment strategies. Numerous clinical studies have underscored the transformative impact of immune checkpoint inhibitors (ICIs) in improving the prognosis of select COAD patients.^24^ Hence, there is a critical need to identify biomarkers associated with ICIs in COAD patients. In this study, we demonstrated that elevated DEPDC1B expression is indicative of a favorable prognosis. DEPDC1B not only regulates the cell cycle but also inhibits the proliferation of COAD cells. Moreover, we observed a positive correlation between DEPDC1B expression and Tfh cell infiltration, a negative correlation with Treg cell infiltration, and positive associations with most immune checkpoints. Additionally, high DEPDC1B expression was indicative of a favorable response to PD‐L1 treatment. These findings highlight the potential of DEPDC1B as a valuable biomarker for predicting outcomes and guiding immunotherapeutic strategies in COAD patients.

Most previous studies have consistently reported upregulated DEPDC1B expression in various cancer types, correlating with poor patient prognosis.^8^, ^9^, ^10^, ^11^, ^12^, ^13^, ^14^, ^15^, ^16^ However, our data present a contrasting perspective, indicating elevated DEPDC1B mRNA levels in COAD patients, with high expression associated with a favorable prognosis. We also observed that COAD patients with elevated DEPDC1B expression exhibited lower EMT and TNM staging. To explore the reasons behind these discrepant findings, we conducted correlation analyses of differentially expressed genes between high and low DEPDC1B expression groups. Our results revealed a positive correlation between DEPDC1B and the expression of CCNB1 and PBK. CCNB1, a crucial member of the cell cycle protein family, promotes the transition from G2 phase to mitosis and has been implicated in the proliferation of colorectal cancer cells.^25^, ^26^ Previous studies have shown that CCNB1 promotes the proliferation of colorectal cancer cells, thus exerting an oncogenic effect.^27^, ^28^ Similarly, PDZ binding kinase (PBK or TOPK) is a serine–threonine mitogen‐activated protein kinase involved in cytokinesis,^29^ and studies have associated it with the growth of colon cancer cells.^30^, ^31^ Intriguingly, despite their known oncogenic roles, COAD patients with high expression of both CCNB1 and PBK exhibited a better prognosis, aligning with the prognostic trend observed for DEPDC1B. Therefore, we should investigate whether DEPDC1B leads to the clinical significance of both by affecting CCNB1 and PBK in a way that is inconsistent with previous studies. Furthermore, our pathway enrichment analysis highlighted an enrichment of the cell cycle pathway in the DEPDC1B high expression group. As DEPDC1B itself is a cell cycle regulatory protein highly expressed in the G2/M phase,^32^ it likely plays a role in regulating cell proliferation.^33^ Experimental results from our study, demonstrating that DEPDC1B knockdown promotes the proliferation of COAD cells, support the hypothesis that DEPDC1B may function as a tumor suppressor in COAD. Overall, our findings suggest a complex and context‐dependent role for DEPDC1B in cancer, urging further exploration of its clinical significance and functional mechanisms in COAD.

The impact of DEPDC1B on the tumor immune microenvironment remains unexplored. Our objective was to investigate whether DEPDC1B functions as a tumor suppressor gene in COAD by influencing the immune microenvironment of tumors. Tumor cells employ various mechanisms to evade immune system recognition, often recruiting and modulating immune cells. Infiltrating Treg cells play a role in exerting immunosuppressive effects on T cells and NK cells,^34^ acting as immunosuppressive cells that tumor cells recruit to enhance resistance to immunosurveillance, thereby promoting tumor growth and expansion.^35^ Conversely, Tfh cells contribute to enhanced anti‐CD8+ T‐cell‐dependent antitumor immunity and anti‐PD‐L1 therapy, preventing tumors from evading the immune response.^36^ A study demonstrated a positive correlation between Tfh‐associated cells and the long‐term survival of colorectal cancer patients.^37^ Our analysis revealed a negative correlation between DEPDC1B expression and the infiltration of Treg cells, along with a positive correlation with Tfh cells. Furthermore, these two immune cell types exhibited differential expression between the high and low DEPDC1B expression groups. This observation provides insight into why patients with high DEPDC1B expression may experience a better prognosis, possibly attributed to the infiltration of these immune cells.

Immunosuppression and evasion of malignant cells are recognized as key characteristics of tumors.^38^ Immunotherapy has rapidly become a cornerstone in cancer treatment, receiving approval from the US Food and Drug Administration (FDA) for various cancer types.^39^, ^40^, ^41^, ^42^, ^43^, ^44^ The predominant immunotherapies target ICIs, focusing on the CTLA‐4 and PD‐1/PD‐L1 pathways. Recently identified immune checkpoints include SIGLEC15,^45^ LAG3,^46^ TIM‐3,^47^ TIGIT^48^ and PD‐L2.^49^ MSI and high TMB have emerged as predictive biomarkers for immunotherapy benefits.^50^, ^51^ Colorectal cancer patients with mismatch repair defects (MSI‐high [MSI‐H]) exhibit positive responses to immunotherapy,^41^ and TMB‐high patients generally have a more favorable prognosis than TMB‐low patients post‐immunotherapy.^52^ Despite these advancements, the role of DEPDC1B in immunotherapy remains unexplored. Our study indicates that patients with high DEPDC1B expression show elevated expression of most immune checkpoints, with positive correlations observed between DEPDC1B and checkpoints such as PD‐L1, CTLA4, and PD‐L2. Additionally, DEPDC1B correlates positively with TMB, and patients with high DEPDC1B expression and TMB‐H demonstrate the best prognosis. Furthermore, DEPDC1B expression is higher in MSI‐H patients. These findings collectively suggest a more favorable response to immunotherapy in patients with high DEPDC1B expression, a prediction validated in the cohort receiving anti‐PD‐L1 treatment. This supports the notion that DEPDC1B holds potential as a target for immunotherapy in COAD.

However, our study has certain limitations. First, the analysis was conducted on the TCGA‐COAD cohort, which has a relatively small sample size, necessitating further validation with a larger dataset. Second, our validation of DEPDC1B's biological functions was confined to the cellular level. Lastly, experimental validation is required to ascertain the role of DEPDC1B in immune cells and its impact on immunotherapy.

## 5 CONCLUSION

In conclusion, our study reveals that, in contrast to its role in other cancers, DEPDC1B may function as a tumor suppressor gene in COAD, impeding the proliferation of COAD cells. The expression of DEPDC1B was found to be positively correlated with Tfh cell infiltration but negatively correlated with Tregs cell infiltration, potentially contributing to the less favorable prognosis observed in patients with high DEPDC1B expression. Moreover, DEPDC1B exhibited predominantly positive correlations with immune checkpoint expression and TMB. Patients with high DEPDC1B expression displayed MSI‐H and exhibited a positive response to PD‐L1 immunotherapy. Therefore, our study tentatively proposes DEPDC1B as a novel immune‐related marker in COAD, potentially serving as a target for immunotherapy.

## AUTHOR CONTRIBUTIONS

**Dandan Zhu:** Formal analysis (equal); writing – original draft (equal); writing – review and editing (equal). **Huolun Feng:** Formal analysis (equal); methodology (equal); writing – original draft (equal). **Zhixiong Zhang:** Investigation (equal); writing – original draft (equal); writing – review and editing (equal). **Jiaqi Li:** Data curation (lead). **Yong Li:** Resources (equal); supervision (equal). **Tieying Hou:** Project administration (lead); supervision (equal).

## FUNDING INFORMATION

This work was supported by the National Natural Science Foundation of China (32370836), the Natural Science Foundation of Guangdong Province (2024A1515012829), the Nanshan District Science and Technology Plan Project (NS2024007), and the Nanshan District Health Major Special Project (NSZD2024023).

## CONFLICT OF INTEREST STATEMENT

The authors declare that they have no competing interests.

## ETHICS STATEMENT

The data we used are from public databases and therefore does not require ethical approval.

## PATIENT CONSENT STATEMENT

Not applicable.

## Supporting information


Figure S1.

Figure S2.

Table S1.

## Data Availability

The datasets analyzed during the current study are available in the TCGA project (https://portal.gdc.cancer.gov/).
