# Supplementary figures and images for: DEPDC1B: A novel tumor suppressor gene associated with immune infiltration in colon adenocarcinoma
Source: Cancer Med. 2024 Aug 1;13(15):e70043. doi: 10.1002/cam4.70043 (PMC11292854; doi:10.1002/cam4.70043)

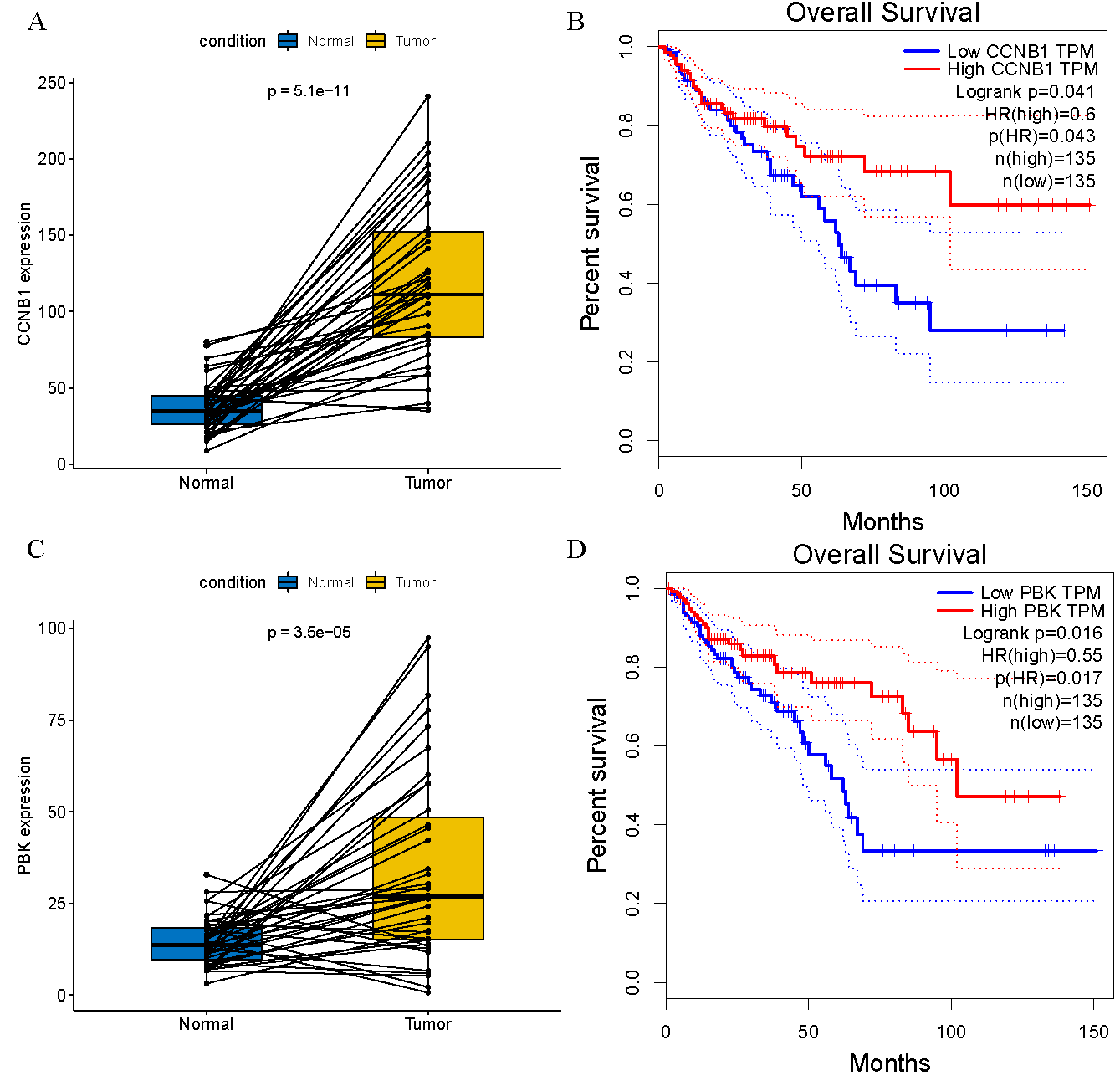

Supplement: Supplementary file 1 — Figure S1. [file CAM4-13-e70043-s001.tif]

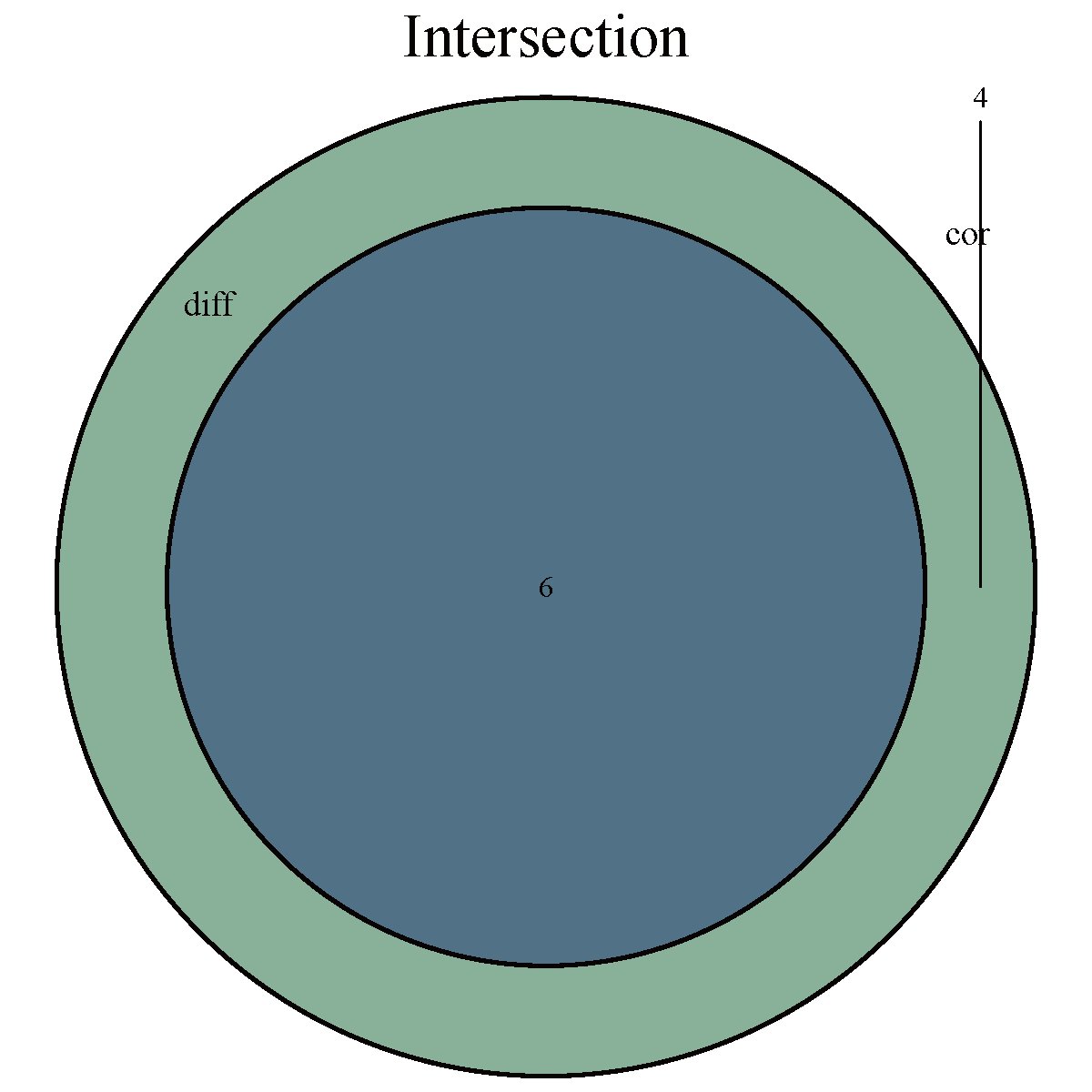

Supplement: Supplementary file 2 — Figure S2. [file CAM4-13-e70043-s002.tif]
