# Supplementary material for: DEPDC1B: A novel tumor suppressor gene associated with immune infiltration in colon adenocarcinoma
Source: Cancer Med. 2024 Aug 1;13(15):e70043. doi: 10.1002/cam4.70043 (PMC11292854; doi:10.1002/cam4.70043)
Supplement: Supplementary file 3 — Table S1. [file CAM4-13-e70043-s003.docx]

| **Primer** | **Sequence (5′–3′)** |
| --- | --- |
| **DEPDC1B-For** | GAGCTACCAGGCTGTGGAAT |
| **DEPDC1B-Rev** | GCCGAAGTTTTGACTGCACC |
| **GAPDH-For** | CTTTGGTATCGTGGAAGGA |
| **GAPDH-Rev** | CACCCTGTTGCTGTAGCC |

**Table S1. The primer sequences used in the qRT-PCR assay.**
